# Global and local perceptual style, field-independence, and central coherence: An attempt at concept validation.

**DOI:** 10.2478/v10053-008-0062-8

**Published:** 2009-04-27

**Authors:** Elizabeth Milne, Marcin Szczerbinski

**Affiliations:** 1 Department of Psychology, University of Sheffield, Western Bank, Sheffield, S10 2TP, UK; 2 Department of Human Communication Sciences, University of Sheffield, 31 Claremont Crescent, Sheffield S10 2TA, UK

**Keywords:** central coherence, perceptual style, global/local perception, field-independence, closure flexibility, visual perception, factor analysis

## Abstract

Historically, the concepts of field-independence, closure flexibility, and weak central coherence have been used to denote a locally, rather globally, dominated perceptual style. To date, there has been little attempt to clarify the relationship between these constructs, or to examine the convergent validity of the various tasks purported to measure them. To address this, we administered 14 tasks that have been used to study visual perceptual styles to a group of 90 neuro-typical adults. The data were subjected to exploratory factor analysis. We found evidence for the existence of a narrowly defined weak central coherence (field-independence) factor that received loadings from only a few of the tasks used to operationalise this concept. This factor can most aptly be described as representing the ability to dis-embed a simple stimulus from a more complex array. The results suggest that future studies of perceptual styles should include tasks whose theoretical validity is empirically verified, as such validity cannot be established merely on the basis of a priori task analysis. Moreover, the use of multiple indices is required to capture the latent dimensions of perceptual styles reliably.

## Introduction

The aim of the present study was to explore the factorial structure of visual perceptual styles. We did this by identifying tasks within the literature that are described as measuring perceptual style and carrying out exploratory factor analysis.

The human visual system excels at object recognition: Objects within the visual scene are identified and perceived as wholes, even when the relevant perceptual data are incomplete. This is demonstrated by the ease with which we can identify familiar objects from incomplete line drawings (Street, 1931), or recognise faces from partially represented black and white forms (e.g., Mooney, 1957). Such abilities are said to be underpinned by a drive for perceptual closure. A related example of closure is seen in contour illusions (e.g., Kanizsa, 1974) in which, due to the organisation of local elements, boundaries and contours are perceived despite not being physically present. This tendency to group features together into a “good form” was identified as the basic law of perception by the Gestalt School of Psychology (the law of *prägnanz*) and highlighted that elements that are proximal to one another or that share a common property (shape, size, colour, orientation, movement in the same direction, etc.) are subject to perceptual grouping. So strong is the tendency to group visual features that it is often difficult to disambiguate constituent local features from a cluttered array. This is demonstrated by difficulty in tasks such as “spot the differences” and is especially effortful when the local features to be detected are embedded within a more complex figure, as in the Embedded Figures Test (Witkin, Oltman, Raskin, & Karp, 1971). Even when the local features are self contained and not embedded in the surrounding context, the perception of the global form still dominates (Navon, 1977).

Despite evidence suggesting a universal bias towards perceptual grouping, and a tendency to perceive the global before the local details, individual variation in the drive for global precedence is also evident. Witkin et al. coined the terms *field-dependence* and *field-independence* (Witkin, Dyk, Faterson, Goodenough, & Karp, 1962) to classify such individual differences. An individual who is field-dependent is highly influenced by the context of the visual scene when processing features, whereas a person who is field-independent is more able to perceive an element independently from its context. The concept of field-(in)dependence was investigated with paradigms such as the Rod and Frame Test (Witkin & Asch, 1948) in which a rod is placed within a tilting frame and participants are required to adjust the rod so that it is upright. This task is accomplished most successfully by participants who can perceive the orientation of the rod independently from the angle of tilt of the frame, that is, participants who are able to resist cues from the surrounding context when making perceptual judgements.

Performance on the Rod and Frame Test correlates highly with that of the Embedded Figures Test (Witkin et al., 1962). The latter is still frequently used in research, and has a range of applications, for example to investigate perceptual style for the purposes of employee psychometric testing (Chapman & Calhoun, 2006), to investigate perceptual styles across cultures (Nisbett & Miyamoto, 2005), and in developmental research, especially in the field of atypical development and autism (Shah & Frith, 1983). Frith (1989, 2003) coined the term *weak central coherence* to describe the clinical and experimental observation that individuals with autism often appear to ignore the (global) aspects of the visual scene that would be most salient to a typical observer, instead showing a tendency to focus on the smaller details, and a reduced ability to integrate material into appropriate context. Since the notion of central coherence was introduced, numerous studies have demonstrated weak central coherence in autism, although the range of tasks used to measure this is varied. For example, children with autism have been shown to succumb to the Gestalt principle of proximity significantly less than controls (Brosnan, Scott, Fox, & Pye, 2004) and to excel at the Embedded Figures Test (Shah & Frith, 1983) and the Block Design sub-test from the WAIS (Shah & Frith, 1993). They also show superior ability to spot differences within two similar visual scenes (Teunisse, Cools, van Spaendonck, Aerts, & Berger, 2001), enhanced ability to detect targets within a visual search array (Jarrold, Gilchrist, & Bender, 2005; Plaisted, O’Riordan, & Baron-Cohen, 1998), a tendency to use a feature based, piecemeal strategy when copying the Rey-Osterrieth Complex Figure (Booth, Charlton, Hughes, & Happé, 2003; Ropar & Mitchell, 2001) and a superior ability to reproduce impossible, but not possible, figures (Mottron, Belleville, & Menard, 1999). There is also some evidence that children with autism may be less susceptible than typically developing children to geometric illusions, such as the Muller-Lyer illusion, and contour illusions such as those formed by the Kanizsa triangle (Happé, 1996, although see Ropar & Mitchell, 1999, and Milne & Scope, 2008, for negative findings). The specific demands of the above tasks are wide ranging, however, and the exact nature of processes that can legitimately be subsumed under a single label of central coherence remains to be established. As the list of studies above illustrates, the term *weak central coherence* is often used to describe tasks that favour local over global processing styles, although this is an extension of the original concept.

 Witkin et al. (1962) reviewed a series of existing correlational and factor analytic studies, and concluded that field-independence was a narrow construct that refers specifically to the “ability to separate an item from its context” (p. 47). In other words, an item must be *embedded* within a structured context rather than merely being surrounded by amorphous material. This early research highlighted that field-independence is separate from the ability to identify an incomplete figure, as measured by Gestalt Completion tests (e.g., Street, 1931; Mooney, 1957). Tasks requiring identification of incomplete figures were only weakly related to those that required dis-embedding, and loaded onto separate factors described as measuring “speed of closure” (Thurstone, 1944). 

 A unique perspective is given by Carroll (1993) who described the structure of human cognitive abilities on the basis of a comprehensive survey and re-analysis of available correlational datasets. The outcome of this analysis with respect to the visuo-spatial domain is summarized in Table 1. Theoretically, Carroll’s position is consistent with Witkin’s as he identified the factor of Closure Flexibility (defined as the speed of disembedding a known stimulus array from a more complex array) which broadly corresponds with Witkn’s notion of field-independence. The operationalisation of the two constructs is somewhat different, however. Whereas Witkin et al. (1962) used the Embedded Figures Test and Block Design to measure field-independence, Carroll demonstrated that closure flexibility is measured with the Embedded Figures task, the Hidden Patterns task and Copying tests, while the Block Design test represents a separate factor of Visualisation. 

**Table 1.** The Visual Perceptual Factors Identified in Carroll’s Systematic Survey of the Factorial Structure of Human Cognitive Abilities

Factors whose existence was reasonably well confirmed through re-analysis of existing datasets.	Definition	Tasks loading highly on the factor
Visualisation	The ability to comprehend imaginary movements in a 3-dimensional space or the ability to manipulate objects in imagination.	Block Design and Object Assembly (WAIS) Block counting tasks Block rotation tasks
Spatial Relation	The ability to perceive spatial patterns or to maintain orientation with respect to objects in space.	Visuo-spatial perspective tasks Card Rotation Task Flags and Figure Rotation
Closure Speed	The ability to combine disconnected, vague visual stimuli into a meaningful whole; to unify an apparently disparate perceptual field into a single concept.	Gestalt Completion Test Street Pictures Closure Test Incomplete Pictures
Closure Flexibility	The manipulation of two configurations simultaneously or in succession. Speed of detecting and dis-embedding a known stimulus array from a more complex array.	Embedded Figures Test Hidden Patterns Test Copying Test
Perceptual Speed	The ability to locate a unique item in a group of identical items. Finding, in a mass of distracting material, a given configuration which is borne in mind during the search.	Cancellation tests Finding “A”s Test Comparison tests
Factors whose existence and/or cognitive interpretation was less well confirmed		
Serial Perceptual Integration	The ability to apprehend and identify a visual pattern when parts of the pattern are presented serially or successively at a high rate.	Tests of integration of successively presented (i.e., motion film) pictorial material.
Spatial Scanning	Speed in visually exploring a wide or complicated visual field.	Maze Tracking speed Map Planning Test
Imagery	Ability to form internal mental representations of visual patterns, and to use such representation in solving spatial problems.	Paper Folding Card Rotation Hands and Bolts
Length Estimation	The ability to compare length of lines or distances.	Shortest Road Test Estimation of Length Test Nearer Point Test
Perception of Illusions	Resistance to illusions involving geometrical figures.	Shape and direction illusion (Poggendorf, Wundt, & Zollner) Overestimation/Underestimation illusions (Muller-Lyer) Size contrast (Delboeuf, Ponzo, & Ebbinghous)
Perceptual Alterations	The rate at which one alternates between ambiguous perceptions.	Retinal rivalry reversals Necker Cube

In sum, the precise conceptual and operational definition of the construct of (weak) central coherence/field-(in)dependence, and its relationship to other dimensions of visual cognition remains unclear. While Carroll’s meta-analysis confirms the existence of such a construct, it suggests a very narrow interpretation: Facility at dis-embedding a known stimulus array from a more complex array, labelled *closure flexibility* and measured primarily with the Embedded Figures Test. This is consistent with Witkin’s definition of *field-independence* but narrower than the notion of *weak central coherence* which, in its research application, if not in Frith’s original formulation, is used to describe a wide variety of tasks that represent a variety of distinct factors within Carroll’s framework.

 Surprisingly, despite the abundance of research on central coherence there has been little attempt to ascertain the degree to which the numerous tests that are currently used to investigate it really do measure the same construct. The research that is available finds little support for a unitary construct. For example, Pellicano, Maybery, and Durkin (2005) carried out a principle components analysis of data collected from 70 children aged between 4 and 5 who performed the Embedded Figures Test, a test of pattern construction (similar to the Block Design subtest from the WAIS), a visuo-motor integration task that required participants to copy and maintain the configuration of a series of images, and a task that required participants to detect target shapes embedded within a complex background (Figure-Ground Test). The analysis produced two factors: one received loadings from the Pattern Construction Task and Visuo-Motor Integration, and the other received loadings from the Embedded Figures Test and the Figure-Ground Test, although the loadings on this factor were not in the expected direction as faster times on the Embedded Figures Test were associated with low scores on the Figure-Ground Test. These data suggest that the four selected tasks do not represent a unitary construct or a coherent index of perceptual style. Another, larger scale study investigated cross-domain perceptual styles in 204 children and adolescents (Booth, 2006). Four low-level visuo-spatial tasks were administered: Embedded Figures Test, Block Design, classification of possible and impossible figures, and a version of the Navon Hierarchical Figures Test. Two principle components were identified. The first received loadings from the Embedded Figures and Block Design tests and was interpreted as a Visual Segmentation Factor. The second received loadings from the Impossible-Possible Figures Test and the Navon Hierarchical Figures Test and was interpreted as a Visual Integration Factor. Higher level tasks such as identification of fragmented pictures, picture memory and drawing style were also administered, however these did not correlate with each other and were not entered into the factor analysis (see Booth, 2006). 

Other authors have reported the strength of correlations between different tasks used to measure perceptual styles, although these correlations are often presented as secondary to the primary hypothesis of the research, which is usually aimed at comparing performance of a developmentally delayed group against a control group. Table 2 presents a summary of these findings. Most, although not all, of this work stems from the field of developmental disorders, specifically autism research, therefore the table specifies whether the data are collected from a clinical or a neuro-typical population.

**Table 2.** A Summary of Reported Correlations and Extracted Factors in Tasks that Measure Perceptual Style

Study	Tests administered	Sample size	Relationships	Pearson’s r coefficients	
				ASD	TD
Booth, 2006^a^	Lower-level tasks				
	Embedded Figures Test (EFT), Block Design (BD), Impossible-Possible Figures Test (I-PFT), Navon Hierarchical Figures (NHF)	ASD = 31, TD = 204	EFT & BD	*r* = .06	*r* = .28**
			EFT & I-PFT	*r* = .01	*r* = -.16*
			EFT & NHF	*r* = .21	*r* = .08*
			BD & I-PFT	*r* = .02	*r* = -.24**
			BD & NHF	*r* = .09	*r* = .03
			NHF & I-PFT	*r* = .10	*r* = .12
	Higher-level tasks				
	Fragmented Pictures (FP), Picture Memory: Description (PM:D), Picture Memory: Recognition (PM:R), Drawing Style (DS)		FP & PM:D	*r* = .21	*r* = -.18*
			FP & PM:R	*r* = .20	*r* = .00
			FP & DS	*r* = -.07	*r* = -.09
			PM:D & PM:R	*r* = .05	*r* = -.01
			PM:D & DS	*r* = -.01	*r* = .00
			PM:R & DS	*r* = -.05	*r* = -.21**
Burnette et al., 2005	Embedded Figures Test (EFT), Block Design (BD), Pattern Construction (PC)	ASD = 23, TD = 20	EFT & PC	*r* = .64**	*r* = .34
			BD & PC	*r* = .58**	*r* = .75**
			EFT & BD	*r* = .28	*r* = .37
Edgin & Pennington, 2005	Embedded Figures Test (EFT), Block Design (BD)	ASD = 24, TD = 34	EFT & BD	†
Jarrold et al., 2005	Children’s Embedded Figures Test (CEFT), Visual Feature Search (FS), Visual Conjunctive Search (CS)	ASD = 18, TD = 18	CEFT & FS	*r* = .80**	*r* = .28
			CEFT & CS	*r* = .29	*r* = .50*
Pellicano, Maybery, Durkin, & Maley, 2006^a^	Pre-school & Children’s Embedded Figures Tests (EFT), Pattern Construction (PC), Figure-Ground Test (F-G), Visual-Motor Integration (VMI)	ASD = 40, TD = 40	EFT & PC	*r* = -.32*	*r* = -.26
			EFT & F-G	*r* = -.28	*r* = -.19
			EFT & VMI	*r* = -.16	*r* = -.28
			PC & F-G	*r* = .22	*r* = -.23
			PC & VMI	*r* = .11	*r* = .47**
			F-G & VMI	*r* = .28	*r* = -.13
Pellicano, Maybery, et al., 2005^a^	Pre-school Embedded Figures Test (PEFT), Pattern Construction (PC), Figure-Ground Test (F-G), Visual-Motor Integration (VMI)	TD = 70	PEFT & PC		*r* = -.31*
			PEFT & F-G		*r* = .11
			PEFT & VMI		*r* = -.06
			PC & F-G		*r* = .03
			PC & VMI		*r* = .47**
			F-G & VMI		*r* = .24*
Ropar & Mitchell, 2001	Children’s Embedded Figures Test (CEFT), Block Design (BD), Selection of illusions including Muller-Lyer (MLI)		BD & CEFT	*r* = -.72**	*r* = -.71**
			CEFT & MLI	*r* = ?, *ns*	*r* = .74**
			BD & MLI	*r* = ?, *ns*	*r* = -.73**
Study	Tests administered	Sample size	Factor loadings^b^		
Booth, 2006	Embedded Figures Test (EFT), Block Design (BD), Impossible-Possible Figures Test (I-PFT), Navon Hierarchical Figures (NHF)	TD = 204	Factor 1: EFT & BD (Visual Segmentation)		
			Factor 2: NHF & I-PFT (Visual Integration)		
Pellicano, Gibson, et al., 2005	Pre-school Embedded Figures Test (PEFT), Pattern Construction (PC), Figure-Ground Test (F-G), Visual-Motor Integration (VMI)	TD = 70	Factor 1: PC & VMI (Visuo-spatial Construction)		
			Factor 2: PEFT & F-G^c^		
Teunisse et al., 2001	Embedded Figures Test (EFT), Children’s Embedded Figures Test (CEFT), Visual Object Spatial Perception-Silhouettes (VOSP-S), VOSP-Object Decision (VOSP-OD), VOSP-Progressive Silhouettes (VOSP-PS), Spot the Differences (SD), Spatial Card Sorting Test (SCST), Wisconsin Card Sorting Test (WCST), Number Card Sorting Test (NCST), California Verbal Learning Test (CVLT), Switch In Series (SIS), Cambridge Neuropsychological Test Automated Battery (CANTAB)	ASD = 35	Factor 1: EFT, CEFT, & SD (Piecemeal Processing)		
			Factor 2: VOSP measures & CVLT (Processing of Meaning)		
			Factor 3: SCST, WSCT, & NCST		
			Factor 4: CANTAB & SIS		
Wasserstein, Barr, Zappulla, & Rock, 2004	Mooney Faces (MF), Street Gestalt Completion Test (SGCT), Street Unstandardised Figures (SUF), Gestalt Completion Test (GCT), Contour Illusion Test (CIT), Facial Recognition (FR)	63 brain injured patients	Factor 1: SGCT, GCT, MF, & CIT (Perceptual Closure)		
			Factor 2: FR		

*Note*. ASD = participants with autistic spectrum disorder. TD = typically developing participants.

a *r* values reflect partial correlations controlling for age and IQ.

b Author’s interpretation, where given, are indicated in parentheses.

c Results were in the opposite direction to that predicted by a unitary construct of weak central coherence.

? = value not given; *ns* indicates that *r* was not given as the relationship was not significant.

† Pearson’s *r* coefficients were not reported, but the relationship between EFT and BD across both groups was significant at *p* < .001.

* *p* < .05.

** *p* < .01.

In general these studies suggest a modest degree of correlation between different tasks purported to measure central coherence, but they do not provide evidence for a single factorial structure. Furthermore, the scope of the analyses was limited as each study typically included only a small subset of tasks.

Since the aim of the present study was to explore the factorial structure of many tasks that have been used to measure either central coherence, field-(in)dependence or global-local perceptual style, we reviewed the literature and identified 14 tasks that are employed for this purpose. Based on this literature review and speculative task analysis, we defined these tasks as measuring the following constructs:

1. The ability to dis-embed and detect a simple stimulus from embedding context (the Embedded Figures Test, the Hidden Patterns Test, and a newly developed Spot the Differences Test).

2. The ability to segment a 2D or 3D shape into individual elements (Block Design and the Copying Test).

3. The ability to detect targets within a non-embedding array (Visual Search).

Note that we made a conceptual distinction between the “embedded” tasks such as the Embedded Figures Test and the Hidden Patterns Test, where targets share contours and boundaries with the embedding context, and the Visual Search Test in which the target is a discrete entity positioned within an array of distractors.

4. The bias towards a more globally or more locally dominated perceptual style (the Navon Hierarchical Figures Test and copying strategy of the Rey-Osterrieth Complex Figure).

5. The ability to draw disparate information into a coherent whole (the Gestalt Completion Test, the Kanizsa task and the Good Form task). 6. The ability to integrate contiguous elements within a single stimulus (Impossible-Possible Figures Test and Muller-Lyer illusion).

7. Global perception without Gestalt demands, when target identification is based solely on the figure’s global form (Silhouettes test from the Visual Object Spatial Perception Battery [VOSP]).

In addition, we measured participants’ sensitivity to coherent motion and coherent form, as significant correlations between performance on the Children’s Embedded Figures Test and coherent motion thresholds have been reported in children with autism (Pellicano, Gibson, Maybery, Durkin, & Badcock, 2005), and detection of both coherent motion and coherent form can be seen as measures of low-level perceptual integration.

## Method

### Participants

Ninety participants, 49 females and 41 males, were recruited to the study via posters displayed on the university campus and an e-mail that was sent to a list of registered volunteers. All participants were students: 60 undergraduates, 7 MPhil students, and 23 PhD students. The average age of the participants was 21 years and 3 months with a standard deviation of 3 years. The exclusion criteria were: speaking a language other than English as a first language and/or being older than 30 or younger than 18. We recruited participants from a range of faculties across the university, the percentage of participants from each faculty was Arts, 21%; Engineering, 14.4%; Medicine, 9%; Law, 7.8%; Pure Science, 25.7%; and Social Science, 20.7%. Participants provided a history of any developmental disorder or existing condition that may affect their performance on the tasks (e.g., uncorrected visual impairment, motor problems, etc.). Four participants disclosed a diagnosis of dyslexia, one was red/green colour blind, and three reported having a lazy eye. These were noted in all cases but as they represent a cross section of the typical population were not considered grounds for exclusion from the study.^1^

### Experimental tasks: “Pen and Paper”

#### The Group Embedded Figures Test (Witkin et al., 1971)

Participants were presented with a booklet of complex figures printed one to a page. Each complex figure had one simple target figure, out of a possible nine, embedded within it. Participants were asked to identify and trace around the simple figure embedded within each complex figure. The test consists of three parts the first of which is considered practice. Parts two and three each contained nine complex figures and had a time limit of 5 min. The test was administered according to the instructions in the manual. The dependent variables were the number of embedded targets identified correctly in parts two and three, out of a possible 18, and the time taken to complete them, out of the total 5 min allowed.

#### Hidden Patterns Test (from the Educational Testing Services Kit; Ekstrom, French, Harman, & Derman, 1976 )

**Figure 1.**
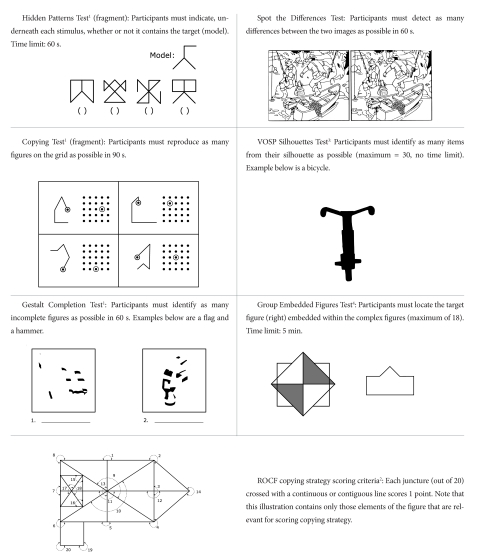
Examples of the stimuli used in the pen and paper tasks. ^1^ Ekstrom, French, Harman, & Derman (1976) Kit of Factor-Refrenced Cognitive Test (KIT) materials are reprinted by permission of Educational Testing Service, the copyright owner. However, the test questions and any other testing information is provided in their entirety by American Psychological Association. No endorsement of this publication by Educational Testing Service should be inferred. ^2^ Adapted from Shorr, Delis, & Massman (1992), from “Memory for the Rey-Osterrieth Figure: Perceptual Clustering, Encoding, and Storage”, *Neuropsychology*, 6, 43-50. ^3^ Reprinted from the Visual and Object Spatial Perception Battery, with permission from Harcourt Assessment. ^4^ Reproduced by special permission of the Publisher, MIND GARDEN, Inc. (www.mindgarden.com) from the GROUP EMBEDDED FIGURES TEST by Herman A. Witkin, Philip K. Oltman, Evelyn Raskin, & Stephen A. Karp. Copyright 1971, 2002 by Herman A.  Witkin et al.. Further reproduction is prohibited without the Publisher’s written consent.

Stimuli were line drawings of geometric patterns. Some of the patterns contained the embedded target configuration. Participants were required to mark, for each item, whether or not the target configuration occurred (see Figure 1). Following an untimed practise session of 10 stimuli, two parts of the test were given. In each part, participants were allowed 60 s to mark whether the target was present or absent in as many patterns as possible. The dependent variable was the number of correct responses given in both parts, out of a possible 200.

#### Gestalt Completion Test (from the Educational Testing Services Kit; Ekstrom et al., 1976)

Drawings composed of black patches representing parts of objects were presented and participants were asked to write down what each drawing depicted (see Figure 1). The experiment was presented in two parts. Following an un-timed practise session participants were given 120 seconds to identify as many objects as they could in each part. The dependent variable was the number of objects identified correctly in both parts, out of a possible 20.

#### Copying Test (from the Educational Testing Services Kit; Ekstrom et al., 1976)

Each item of this task consisted of a four-line geometrical figure and a square matrix of dots presented in a 5 x 5 array. The task was to copy the figure exactly onto the matrix of dots (see Figure 1). Again this task was administered in two parts. Following an untimed practise session, participants were given 90 s to copy as many patterns as they could in each part. The dependent variable was the number of correctly copied figures, out of a possible 64.

#### VOSP-Silhouettes(Warrington & James, 1991)

The stimuli of this task were drawings of 30 objects presented as silhouettes. Each participant had unlimited time to identify each object. The dependent variable was the number of objects correctly identified, out of a possible 30.

#### Spot the Differences Test

This was a traditional spot the difference puzzle adapted specifically for this study. The stimuli originally appeared in a pre-1990 edition of a Polish popular weekly magazine called *Przekrój*. These images were chosen as they were considered to be sufficiently challenging for adults and were highly unlikely to have been seen previously by any of the participants. Two versions were given: a kitchen scene and a fishing scene (see Figure 1). Each scene was presented as a black and white line drawing reproduced twice on one piece of A4 paper. The participant was informed that the two pictures differed in a number of small details, and were asked to mark any differences they detected by putting a cross in the appropriate place on the right-hand side image. A 60 s time limit was given for each picture. The differences could concern placement of features, size of features, number of clustered features, orientation of features, and addition/subtraction of features. The order of scene presentation (kitchen or fishing) was counterbalanced between participants. The dependent variable was the total number of differences detected, out of a possible 39 and 34 for the kitchen and fishing scenes, respectively.

#### Rey-Osterrieth complex figure (Rey figure)

 Participants were presented with a laminated card showing the Rey figure (Osterrieth, 1944), given a blank piece of paper and a pencil, and asked to reproduce the figure. Following a break of 5 min during which they engaged in another, unrelated task, they were given a surprise recall test and asked to re-draw the figure on a new piece of paper from memory. Participants were filmed in both conditions. The copies were scored for accuracy and strategy, recall was scored for accuracy only. Accuracy was scored according to Osterrieth’s (1944) criteria, adapted by Taylor (1959; reproduced in Lezak, Howieson, Loring, Hannay, & Fischer, 2004, p. 542) which identifies 18 elements of the figure. Ambiguous cases were resolved using recommendations made by Strupczewska (1990), who further elaborated the Osterrieth’s scoring criteria and provided examples. A maximum of 2 points was available for the reproduction of each element, giving a maximum possible score of 36. Strategy was scored by adopting the criteria suggested by Shorr, Delis, and Massman (1992) who considered the Rey figure as an assembly of eight sub-components. For each sub-whole, junctures were identified where breaks in continuous drawing of the sub-wholes can occur. Participants received 1 point for every juncture that was completed by either continuous or contiguous lines, with a maximum possible of 20. A high score on this system therefore indicates a globally biased drawing style, whereas a low score indicates a more locally biased, piecemeal drawing style. The scoring system template is illustrated in Figure 1. In total, three dependent variables were obtained from this test: copy accuracy score, recall accuracy score, and strategy score. 

### Experimental tasks – computerised

The following computerised tasks were presented on a Viglen laptop computer, the screen of which was 1024 pixels wide (285 mm) and 768 pixels high (215 mm), which refreshed at 60 Hz. The experiments were written and presented in either E-prime (Psychology Software Tools, Inc.; www.pstnet.com) or Visual Basic (Visual Studio 2005, www.microsoft.com). Luminance of the stimuli and background were measured with a Sekonic dual spot (1° photometer) and Michelson contrast of the stimuli was calculated with the following formula (*Lmax* -­ *Lmin*)/(*Lmax* + *Lmin*). The visual angle of the stimuli below is calculated based on an assumed distance of 47 cm from the computer screen.

#### Hierarchical Figures Test (based on Navon, 1977)

##### Stimuli

**Figure 2.**
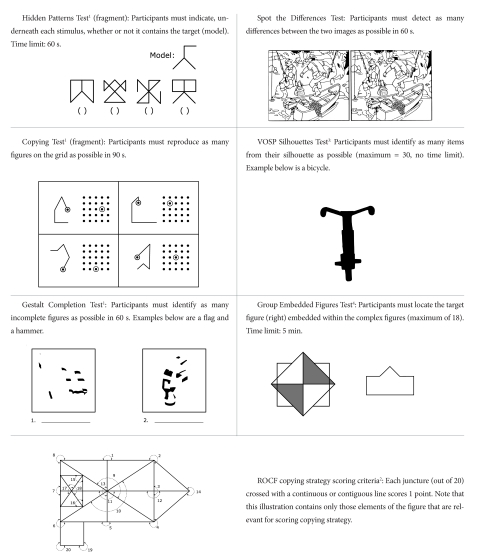
Examples of the stimuli used in the computer tasks. ^1^ Reproduced with permission from *The British Journal of Developmental Psychology* © The British Psychological Society.

Hierarchical stimuli consisted of large “global” letters composed of smaller “local” letters. Target stimuli were either “H” or “S” and neutral letters were “X”. The stimuli were compatible, neutral, or incompatible depending on the pairing of target and distractor stimuli and are detailed in Figure 2. All stimuli were black and were presented on a grey background (Michelson contrast = 76%). The global outline of the stimuli subtended 3.66° x 4.87°.

##### Design and procedure

A selective attention design was used, whereby participants were instructed to indicate via a two-alternative choice key press whether the letter at the designated level was “H” or “S”. A total of 144 trials were presented in 12 blocks. In half of the blocks the participant was instructed to identify the letter at the global level and in the other half the letter at the local level. Within each block the three different stimulus types: compatible, neutral, or incompatible, and were presented randomly but equiprobably. To discourage the participant from looking at a fixed point on the screen where “local” letters always appeared, the stimulus appeared randomly either below or above a fixation point that remained on the screen for 500 ms. Each stimulus stayed on the screen for 150 ms, and was replaced by a pattern mask that remained until the participant made a response, following which the screen went blank for 500 ms before presentation of the next fixation point. Response time and accuracy were recorded.

#### Muller-Lyer Line Length Illusion Task

##### Stimuli

Six stimulus pairs were created. Each consisted of one horizontal line with illusion inducing fins placed above another parallel line without fins. The upper line with fins always subtended 7.3°. The lower line subtended either 7.3°, 6.9°, or 7.7°; the length of the lower line was manipulated so that it was shorter than, longer than, or the same length as the upper line. The task was to indicate via a three-alternative choice key press whether the lower line was longer than, shorter than or the same length as the upper line. In three of the stimulus pairs the upper line had fins that pointed inwards and in the other three stimulus pairs the fins on the upper line pointed outwards. In the non-illusory condition, the lower line looked, and really was, either longer or shorter than the upper line with fins. In the illusory condition, the lower line was either longer or shorter than the upper line with fins but looked the same length as the upper line, or the lower line was the same length as the upper line with fins but looked either longer or shorter depending on the fins of the upper line. Examples of illusory (upper line) and non-illusory (lower line) are presented in Figure 2. The stimuli were black against a white background (Michelson contrast = 87%).

##### Design and procedure

Each stimulus pair was presented eight times (*N* = 48 trials) in random order. Prior to stimulus presentation a central fixation cross appeared for 500 ms. The stimulus remained on the screen until the participant made a response, following which the screen went blank for 500 ms before presentation of the next fixation cross. Response time and accuracy were recorded.

#### Kanizsa Illusory Contour Task (based on Ringach & Shapley, 1996)

##### Stimuli

Stimuli were illusory rectangles induced by white “pac-man” figures presented on a black background. The dimensions of the stimuli were governed by the angle of pac-man rotation. In half of the images the pac-man figures were rotated to create the perception of a “fat” rectangle while in the other half they were rotated to create the perception of a “thin” rectangle. The degree of rotation was either 5º, 10º, or 15º from the horizontal midline, this resulted in a percept with varied degrees of “fatness” or “thinness”. Participants were instructed to identify whether the shape was fat or thin via a two-alternative choice key press. The images were presented at different orientations: straight, rotated 45º to the left, or 45º to the right to prevent any participant using a strategy of ascertaining the shape of the induced rectangle by looking at the angle of orientation of one inducer only. Each stimulus subtended 5.48º × 8.52º. Control, non-illusory stimuli were created that were identical to the illusory stimuli apart from white line contours (2 pixels wide) that were drawn to highlight the rectangle. Michelson contrast of the stimuli was 87%.

##### Design and procedure

Two separate blocks of trials were administered: the illusory block and the control block. The order of block presentation was counterbalanced between participants. In each block, the six stimuli were presented nine times giving a total of 54 trials per block. Each stimulus was equally likely to be presented upright, oriented to the left, or oriented to the right. Prior to stimulus presentation a central fixation cross appeared for 500 ms. The stimulus remained on the screen until the participant made a response, following which the screen went blank for 500 ms before presentation of the next fixation cross. Response time and accuracy were recorded.

#### Visual search for a conjunctive target (based on Plaisted et al., 1998>)

##### Stimuli

The stimuli consisted of an array of letters in a virtual grid, from which participants were instructed to search for a target amidst distractors. The target was a red “X”, the distractors were red letters “T” and green letters “X”. Each letter measured approximately 0.5° x 0.5° and the virtual grid subtended 20.3° x 20.3°.

##### Design and procedure

Participants were instructed to press one of two keys to indicate whether the target was present or not. From a total of 60 trials, 30 contained the target. In each trial 5, 15, or 25 distractors were presented with equal probability but random selection. Prior to the array presentation, a fixation cross appeared on the screen for 500 ms, and disappeared once the stimulus appeared. The stimulus remained on the screen until the participant made a response, following which the screen went blank for 500 ms before presentation of the next fixation cross. Response time and accuracy were recorded.

#### Impossible-Possible Figures Test

##### Stimuli

 The stimuli were figures used by von Karolyi, Winner, Gray, and Sherman (2003), and were adapted from the set of possible and impossible images developed by Schacter, Cooper, and Delany (1990). These were geometrically possible (*n* = 12) or impossible (*n* = 11) black line drawings presented on a white background (Michelson contrast = 87%). Participants indicated via a two-alternative choice key press whether the presented stimulus was geometrically possible or impossible. Each stimulus subtended approximately 5.1° x 5.5°. 

##### Design and procedure

Prior to the presentation of each figure, a fixation cross appeared on screen for 500 ms. One of the 23 figures then appeared at random and remained on the screen until a response was made, following which the screen went blank for 500 ms before the reappearance of the fixation cross. Response time and accuracy were recorded.

#### Good Form Task (adapted from Williams & Bologna, 1985)

##### Stimuli

“Good form” experimental stimuli were pairs of vertical brackets that were designed to elicit perceptual closure. “Poor form” control stimuli were horizontal and vertical brackets that did not elicit perceptual closure (see Figure 2. In each block participants were instructed to sort the stimuli into two groups (arbitrarily classified as left or right) via a two-alternative choice key press. Theoretically this task could be successfully completed by selectively attending to the right bracket of each pair only. The experimental images subtended 1.2° x 1.8°, the control images subtended 3.65° x 1.8°.

##### Design and procedure

 In each trial, one bracket pair appeared on the screen; 144 trials were organised into two conditions (experimental good form and control poor form) and six separate blocks. Four out of six blocks were defined as “simple” as only one stimulus pair was associated with each response. In the simple blocks the irrelevant (left) bracket was predictable, that is it always faced the same way. The other two blocks were defined as “orthogonal” as two stimulus pairs were associated with each response. In these blocks the irrelevant left bracket was unpredictable, in other words, it could face either direction. Williams and Bologna (1985) found that reaction time to classify the stimuli was significantly longer in the orthogonal experimental blocks than in the simple experimental blocks or any control blocks. They interpreted this as a result of perceptual grouping, that is the greater the tendency for perceptual grouping the harder it was to selectively attend to the relevant right bracket (and ignore the irrelevant left) to the detriment of performance. Since the aim of this task was to test the drive for perceptual grouping rather than memory, visible prompts were available at all times to remind participants which stimulus pairs were associated with which response. The order of block administration was counterbalanced across participants. After instruction, a fixation cross appeared on the screen for 500 ms, this was replaced by the stimulus which remained on the screen until a response was made, following which, the screen went blank for 500 ms before presentation of the next stimulus. Response time and accuracy were recorded. 

### Additional psychometric variables

#### Choice RT

As most of the experimental tasks administered above required participants to make an alternative choice by pressing one of two keys as quickly as possible, this control task provided a baseline measure of choice reaction time.

##### Stimuli

The stimuli were red and blue circles with a diameter of 5.36°. They appeared in the centre of the computer screen against a white background (Michelson contrast = 58% and 52%, respectively).

##### Design and procedure

The circles were presented in random order. Participants were instructed to indicate the colour of the stimulus via a two-alternative choice key press. Prior to the presentation of each stimulus a fixation cross appeared on the screen for 500 ms, the stimulus then appeared and remained until a response had been made, after which the screen went blank for 500 ms before the reappearance of the fixation cross. Each participant completed 32 trials. Response times and accuracy were recorded.

#### Motion coherence threshold (Hansen, Stein, Orde, Winter, & Talcott, 2001)

A standard random dot kinematogram (RDK) stimulus was used, consisting of two horizontally adjacent panels of moving dots. Each contained 300 white dots (1 pixel) of high contrast (approx. 90%) against a black background. Each panel was rectangular, subtending 10º × 14º and separated horizontally by 5º. One panel contained a variable proportion of target dots that moved coherently to the left and right over successive screen refreshes, whilst the remaining noise dots in the panel moved with the same speed but in a direction that randomly changed between refreshes. The other panel contained only noise dots. To prevent tracking of individual dots, the lifetime for each dot was fixed at three animation frames (85 ms) after which time the dot was regenerated at a random position inside the same panel.

#### Form coherence threshold (Hansen, Stein, Orde, Winter, & Talcott, 2001)

Two rectangular panels were presented side by side, matched in size and overall luminance to the motion task. Each panel consisted of 600 short, high contrast line elements, with each element being 0.4º in length. In one panel there was a coherent form signal, defined by line elements that were oriented tangentially to imaginary concentric circles within an area of 8º diameter. Signal coherence was varied by modifying the percentage of aligned elements. At 100% coherence therefore, all line elements within the 8º boundary would be perfectly aligned. Elements outside the 8º area were orientated randomly. In the other panel, all elements were randomly orientated.

In both the motion and form coherence tasks participants were asked to identify the patch that contained the coherent signal via two alternative forced choice key press. Auditory feedback was given after each trial. Three sets of trials in each task were administered. The final threshold for each task was calculated as the average threshold of the three sets of trials. Signal coherence was varied by modifying the number of coherent elements within the target patch. Each set of trials started with signal at 75% coherence. Following a correct response, coherence decreased by 1.5 dB and following an incorrect response coherence increased by 0.5 dB (Kaernbach, 1991). Each set was terminated after 10 reversals. Threshold within each set was calculated as the geometric mean of the last 8 reversal points. The order of presentation of form and motion coherence tasks was counterbalanced between participants.

#### General intellectual ability

IQ was assessed with the Wechsler Abbreviated Scales of Intelligence (WASI; Wechsler, 1999). This consists of four subtests: two verbal (Vocabulary and Similarities) and two performance (Block Design and Matrix Reasoning).The tasks were administered according to the manual. As the Block Design subtest is also an index of perceptual styles this was included as one of the experimental variables and IQ was derived from two subtests only (Vocabulary and Matrix Reasoning), using norms provided in the test manual.

### General procedure

The study received ethical approval from university ethics sub-committees. All participants provided informed consent before taking part and received £10 for their participation. The tasks were administered during two sessions (at least one day apart) that lasted approximately 2 hours each. Four different schedules counterbalanced the order of task administration. Three participants did not return for the second session.

## Results

### Preliminary analysis

Descriptive statistics for all tasks are presented in Appendix A. In order to establish whether the tasks used in this study produced within-task patterns of results that were consistent with those reported in published studies, preliminary analyses were carried out and are reported in Appendix B. In all cases median response times based on correct responses only are reported and analysed. There were some missing data points for some variables (reflected in the varying degrees of freedom). All of the tasks showed the expected pattern of results based on previously published studies (see Appendix B).

### Selection of tasks for correlation and factor analyses

The following tasks were selected for factor analyses: (a) Block Design raw score, (b) Group Embedded Figures Test (accuracy and completion time), (c) Copying Test accuracy, (d) Gestalt Completion Test accuracy, (e) VOSP-Silhouettes accuracy, (f) Spot the Differences Test accuracy, (g) Rey figure copying strategy, (h) Impossible-Possible Figures Test (median RT [reaction time] to classify the impossible figures), (i) Navon Hierarchical Figures Test (accuracy and median RT to correctly identify incompatible targets separately at the global and local level), (j) Muller-Lyer (number of illusions correctly identified and median RT to correctly identify them), (k) Kanizsa (number of shapes defined by illusory contours correctly identified and median RT to identify them; Kanizsa, 1974), (l) Visual Search Task (number of targets detected during visual search amongst 25 distractors and median RT to detect them), and (m) Good Form Task (the experimental orthogonal condition, median RT to correctly classify the brackets). These tasks, or parts of tasks, were included because they tap most directly into the constructs of global and local processing under investigation.

The following tasks or conditions were excluded because they were considered to be control tasks and as such did not tap global/local perception directly: (a) Rey Figure accuracy of copy and recall, the possible figures (control) condition of the Impossible-Possible Figures Test; (b) Navon Hierarchical Figures accuracy and RT in the compatible and neutral conditions; (c) Muller-Lyer non-illusory condition; (d) Kanizsa non-illusory condition; (e) Visual Search Task 5 and 15 distractors conditions; (f) all control conditions and experimental simple conditions of the Good Form Task. Accuracy scores the Impossible-Possible Figures and Good Form tasks were also excluded as most participants obtained ceiling scores. Additional variables – IQ (as measured by WASI), choice RT, and Form and Motion Coherence thresholds – were not entered into factor analyses, but used in correlation analyses (reported below).

### Reliability analyses

Indices of reliability were computed for all measures entered into correlation and factor analyses, with the exception of the WASI IQ variables whose psychometric properties are well described in the literature. Measures of split-half, parallel test, and internal consistency reliability were computed, as appropriate. For the Rey figure copying strategy, two indices were obtained: internal consistency (based on data from all participants, scored and agreed jointly by both authors) and inter-rater reliability (using data from 30 participants, scored independently by a person who was blind to the authors’ scores). The results are presented in Table 3.

**Table 3.** Reliability of the Measures Used in the Study

Task	Reliability	Reliability index
Group Embedded Figures Test		
RT	.568	Equal length Spearman-Brown
Accuracy	.853	Equal length Spearman-Brown
Hidden Patterns Test		
Accuracy	.863	Equal length Spearman-Brown
Gestalt Completion Test		
Accuracy	.422	Equal length Spearman-Brown
Copying Test		
Accuracy	.854	Equal length Spearman-Brown
VOSP-Silhouettes		
Accuracy	.594	Cronbach’s alpha
Spot the Differences Test		
Accuracy	.522	Equal length Spearman-Brown
Rey figure		
Copying strategy: Internal consistency	.850	Cronbach’s alpha
Copying strategy: Inter-rater reliability	.962	Intraclass correlation
Navon Hierarchical Figures Test, incompatible condition		
Global RT	.937	Equal length Spearman-Brown
Global accuracy	.459	Equal length Spearman-Brown
Local RT	.858	Equal length Spearman-Brown
Local accuracy	.583	Equal length Spearman-Brown
Muller-Lyer illusory condition		
RT	.950	Equal length Spearman-Brown
Accuracy	.767	Equal length Spearman-Brown
Kanizsa illusory condition		
RT	.931	Equal length Spearman-Brown
Accuracy	784	Equal length Spearman-Brown
Visual Search, target present amongst 25 distractors		
RT	.855	Equal length Spearman-Brown
Accuracy	.153	Equal length Spearman-Brown
Impossible-Possible Figures Test		
RT	.909	Equal length Spearman-Brown
Good Form task experimental orthogonal block		
RT	.925	Equal length Spearman-Brown
Choice RT		
RT	.909	Equal length Spearman-Brown
Motion coherence (% threshold)	.818	Cronbach’s alpha
Form coherence (% threshold)	.567	Cronbach’s alpha

The reliability of the tests varied considerably. The tests with relatively low reliability (below .70) were typically the measures of accuracy rather than response time. The lowest reliabilities (below .50) were obtained for Visual Search accuracy, Gestalt Completion Test accuracy, and Navon Hierarchical Figures Test accuracy in the global incompatible condition. This may result from the fact that two of the tests were relatively easy (Visual Search and Navon global incompatible condition, see Appendix A), and two of them (Visual Search and Gestalt Completion Test) were relatively short.

### Relationships between tasks

#### Data preparation

Some data points were missing due to equipment failure, administrators’ errors, or participants’ failure to attend one of the assessment sessions. For most variables one to four data points were missing, which constituted 1.1 - 4.4% of potentially available data. The only exceptions were Rey figure strategy and Visual Search (accuracy and reaction time) with 15 (16.7%) and 9 (10%) data points missing, respectively. The missing data points were replaced using expectation maximization (EM) method (Tabachnick & Fidell, 2001). The EM procedure included all cognitive variables and WASI raw scores. Little’s MCAR test was carried out on all variables and was not significant (χ^2^ = 445.724, *df* = 452, *p* = .574) indicating that data can be assumed to be missing at random.

All variables that were entered into correlation and factor analyses were winsorised for outliers: All scores that were more than 2.33 standard deviations away from the mean (which, under normal distribution, corresponds to the top and bottom 1% of cases) were set to the value of 2.34 standard deviation away from the mean. After this treatment, no variables showed extreme departures from normality (defined as the absolute value skewness greater than 2 and/or the absolute value of kurtosis greater than 7; West, Finch, & Curran, 1995, as cited in Fabrigar, Maccallum, Wegener, & Strahan, 1999). The largest skewness (1.46) was observed on the Impossible Figures RT data, and the largest kurtosis (1.91) on Kanizsa accuracy.

#### Correlation analysis

Table 4 reports zero-order and partial correlations, controlling for estimated full scale IQ based on two subtests from the WASI, and choice RT, for all variables. It is apparent that the significant correlations appear mostly between non-computerised tasks. Also, the differences between zero-order and partial correlations are mostly negligible, suggesting the relationships between variables in the study are not mediated by general cognitive ability or choice reaction time.

**Table 4.** Correlations Between the Variables

	Block Design	Embedded Fig.. acc.	Embedded Fig. RT	Hidden Patterns	Gestalt Completion	Copying	Silhouettes	Spot the Differences	Rey figure Strategy	Navon Global acc.	Navon Global RT	Navon Local acc.	Navon Local RT	Muller-Lyer acc.	Muller-Lyer RT	Kanizsa acc.	Kanizsa RT	Visual Search acc.	Visual Search RT	Impossible. Figures RT	Good Form RT	Motion Coherence	Form Coherence	Choice RT	WASI IQ (2 subtests)
Block Design	-	.55**	-.44**	.37**	.40**	.54**	.36**	.42**	.35**	.10	-.06	.40**	-.03	.16	-.14	.24*	-.08	.19	-.28**	.00	-.48**	-.27**	-.25*	-	-
Embedded Fig. acc.	.57**	-	-.50**	.28**	.07	.28**	.12	.40**	.22*	.14	-.05	.29**	-.03	.20	-.03	.25*	-.07	.07	-.13	.15	-.23*	-.18	-.25*	-	-
Embedded Fig. RT	-.45**	-.51**	-	-.26*	-.23*	-.36**	-.18	-.29**	-.04	.01	.18	-.28**	.18	-.14	.11	-.10	.10	-.06	.16	.03	.19	.14	.00	-	-
Hidden Patterns	.39**	.30**	-.26*	-	.14	.28**	.15	.20	.12	-.16	-.22*	.16	-.17	.24*	-.22*	.03	-.11	.03	-.33**	-.06	-.15	.00	-.17	-	-
Gestalt Completion	.42**	.10	-.24*	.16	-	.25*	.14	.22*	.21*	.16	-.01	.26*	-.02	.23*	.06	.16	-.03	.01	-.04	.07	-.17	.01	-.08	-	-
Copying	.51**	.27*	-.36**	.27*	.24*	-	.19	.24*	.09	.02	.04	.14	-.04	.13	-.07	.12	-.02	.20	-.13	-.08	-.29**	-.19	-.10	-	-
Silhouettes	.39**	.15	-.21*	.18	.17	.18	-	.18	.24*	.01	-.15	.20	-.03	.06	-.16	.08	-.12	.06	-.14	-.14	-.20	-.27**	-.04	-	-
Spot the Differences	.42**	.40**	-.29**	.20	.23*	.24*	.18	-	.19	.27*	.05	.22*	.06	.11	.14	.43**	-.18	.16	-.10	.19	-.19	-.14	-.18	-	-
Rey figure Strategy	.31**	.19	-.02	.11	.19	.08	.18	.19	-	.15	.09	.24*	.22*	.02	-.06	-.05	-.02	.04	-.08	.01	-.21*	.01	-.37**	-	-
Navon Global acc.	.10	.14	.00	-.17	.16	.03	.03	.26*	.14	-	.22*	.13	.36**	-.03	.09	.25*	-.04	.15	-.05	.26*	-.06	-.04	-.01	-	-
Navon Global RT	-.11	-.07	.16	-.25*	-.04	.07	-.12	.02	.05	.23*	-	.18	.57**	-.06	.28**	.00	.09	.10	.30**	.29**	.10	-.07	-.15	-	-
Navon Local acc.	.41**	.30**	-.29**	.17	.27*	.13	.22*	.22*	.22*	.13	.17	-	-.13	.17	-.10	.12	-.03	.05	-.20	.09	-.33**	-.23*	-.21*	-	-
Navon Local RT	-.06	-.04	.15	-.19	-.04	-.01	.02	.03	.16	.37**	.63**	-.11	-	-.06	.36**	.09	.08	.13	.07	.27*	.18	-.01	-.18	-	-
Muller-Lyer acc.	.19	.22*	-.15	.26*	.25*	.12	.09	.11	.01	-.04	-.10	.18	-.09	-	.19	.31**	.06	.14	-.13	.02	.04	-.09	-.13	-	-
Muller-Lyer RT	-.10	-.01	.09	-.20	.08	-.07	-.12	.14	-.06	.08	.23*	-.09	.32**	.21	-	.25*	.24*	.24*	.33**	.26*	.31**	-.02	-.04	-	-
Kanizsa acc.	.28**	.28**	-.12	.07	.19	.10	.13	.43**	-.07	.24*	-.05	.14	.05	.33**	.26*	-	-.19	.22*	-.13	.08	.03	-.11	-.13	-	-
Kanizsa RT	-.12	-.09	.10	-.14	-.06	.01	-.11	-.20	-.04	-.02	.19	-.03	.18	.02	.20	-.22*	-	.07	.16	-.03	.25*	.01	.26*	-	-
Vis. Search acc.	.20	.08	-.06	.04	.02	.20	.07	.17	.03	.15	.08	.05	.12	.14	.25*	.22*	.06	-	-.03	-.10	.06	-.27*	-.11	-	-
Vis. Search RT	-.30**	-.14	.16	-.34**	-.06	-.11	-.15	-.11	-.09	-.04	.33**	-.20	.12	-.15	.31**	-.15	.21	-.03	-	.08	.10	.01	.12	-	-
Impossible Fig. RT	.00	.15	.02	-.06	.07	-.08	-.11	.18	-.01	.27*	.30**	.10	.28**	.02	.26*	.08	-.01	-.10	.09	-	.16	.12	-.17	-	-
Good Form RT	-.43**	-.20	.15	-.15	-.16	-.25*	-.10	-.19	-.25*	-.02	.20	-.29**	.29**	.02	.29**	.02	.31**	.06	.14	.18	-	.05	.16	-	-
Motion Coherence	-.26*	-.18	.15	.00	.01	-.19	-.28**	-.13	.03	-.05	-.10	-.24*	-.06	-.08	-.02	-.10	-.03	-.26*	-.01	.10	-.01	-	.10	-	-

*Note*. Zero-order correlations are below the diagonal (*df* = 90). Partial correlations controlling for 2-subtests WASI IQ and choice RT are above the diagonal in italics (*df* = 86). *p < .05. two-tailed. **p < .01, two-tailed.

#### Factor analyses

The factor analyses reported below used the Alpha Factoring method of factor extraction. This method was chosen because it “considers the variables in the analysis to be a sample from the universe of potential variables” (SPSS, 2005). This was appropriate in our case, the “universe” being all potential variables measuring perceptual style. Alpha factoring also maximises the reliability (internal consistency) of the extracted factors. This results in a conservative estimate of the proportion of total variance explained by the latent factors. Factors were rotated using the Direct Oblimin method.

 Preliminary analyses examined the suitability of the data for factor analysis which followed the recommendations of Field (2005). Bartlett’s test of sphericity was highly significant (χ^2^ = 514.4, *df* = 210, *p* < .001), indicating some relationships existed between variables, which makes the correlation matrix suitable for factor analysis. Determinant of correlation matrix was .002 (well above the recommended minimum value of .00001) indicating that multicollinearity was not a problem. On the other hand the Kaiser-Meyer-Olkin measure of sampling adequacy was .653, which is above the minimum recommended value of .500 yet “mediocre” (Kaiser, 1974, as cited in Field, 2005). This indicates that the pattern of correlations was relatively diffuse, making it relatively hard to extract distinct and reliable factors. The analysis of anti-image correlation matrix diagonals, which indicate sampling adequacy of individual variables, brought unsatisfactory results (< .50) for the Navon incompatible global RT. This variable was excluded from the analysis, which resulted in the improvement of sampling adequacy (Kaiser-Meyer-Olkin = .699).

Seven factors were extracted using Kaiser’s criterion of eigenvalues greater than one. Together they explained 44.8% of total variance. Most communality values were lower than .50; that is the seven latent factors could typically account for less than half of the variance of individual variables. Only Block Design showed high communality values (above .80, see Table 5). The pattern matrix, representing coefficients of regression of variables on factors, is displayed in Table 6, while the structure matrix, representing factor loadings (coefficients of correlations between variables and factors), is displayed in Table 7.

**Table 5.** Communalities in the First Factor Analysis

Variables	Initial	Extraction
Block Design	.701	.830
Group Embedded Figures Test accuracy	.408	.474
Group Embedded Figures Test RT	.534	.543
VOSP-Silhouettes	.228	.176
Gestalt Completion Test	.342	.326
Hidden Patterns Test	.330	.381
Copying Test	.341	.359
Spot the Differences Test	.398	.460
Rey figure: Copying strategy	.262	.282
Impossible Figures RT	.291	.508
Muller-Lyer illusory condition accuracy	.285	.445
Muller-Lyer illusory condition RT	.425	.618
Visual Search, target present within 25 distractors accuracy	.220	.277
Visual Search, target present within 25 distractors RT	.282	.397
Kanizsa illusory condition accuracy	.438	.689
Kanizsa illusory condition RT	.263	.334
Good Form experimental orthogonal RT	.445	.559
Navon Hierarchical Figures Test, incompatible, global acc	.336	.378
Navon Hierarchical Figures Test, incompatible, local acc	.290	.312
Navon Hierarchical Figures Test, incompatible, local RT	.374	.608

**Table 6.** Pattern Matrix of the First Factor Analysis

Variables	Factors						
	1	2	3	4	5	6	7
Embedded Figures acc	.715						
Embedded Figures RT	-.687						
Block Design	.537					.415	
Copying	.468						
Navon local RT		.771					
Navon global acc		.501					
Muller-Lyer acc			.648				
Kanizsa acc			.455	-.597			
Kanizsa RT				.504			
Spot the Differences	.370			-.379			
Impossible Figures RT					.574		
Visual Search acc					-.397		
Gestalt Completion						.485	
Rey figure strategy						.480	
Good Form RT		.313		.309		-.461	
Navon local acc						.416	
VOSP-Silhouettes							
Visual Search RT							-.603
Muller-Lyer RT			.465				-.508
Hidden Patterns							.402

*Note*. Coefficients are sorted by size, those lower than .30 are not displayed.

**Table 7.** Structure Matrix of the First Factor Analysis

Variables	Factors						
	1	2	3	4	5	6	7
Block Design	.779				-.334	.689	.351
Embedded Figures acc	.711						
Embedded Figures RT	-.672						
Copying	.514				-.359		
Spot the Differences	.509			-.495		.316	
Navon local RT		.751					
Navon global acc		.534					
Muller-Lyer acc			.624				
Kanizsa acc			.549	-.631			
Kanizsa RT				.504			
Impossible Figures RT		.379			.561		
Visual Search acc					-.404		
Good Form RT		.303		.370		-.537	
Navon local acc	.372					.497	
Gestalt Completion	.302					.489	
Rey figure strategy						.487	
VOSP-Silhouettes						.326	
Visual Search RT							-.602
Muller-Lyer RT		.378		.515			-.588
Hidden Patterns	.414						.497

*Note*. Factor loadings are sorted by size, those lower than .30 are not displayed. Loadings that are statistically significant according to Stevens' (1992) interpretation are in bold.

We interpreted the seven factors as follows:

Factor 1: Dis-embedding. This factor received six substantial(greater than .40) factor loadings, three of which (Block Design, Embedded Figures accuracy, and RT) were significant according to Stevens’ (1992, p. 394) criteria for testing the statistical significance of factor loadings, which imply the critical value of .534 for *N* = 90. Out of the six variables loading substantially on Factor 1, four (Embedded Figures accuracy and RT, Copying Test and Hidden Patterns Test) represent Carroll’s (1993) Closure Flexibility factor, defined as “speed of detecting and *disembedding* a known stimulus array from a more complex array” (p. 341). The fifth variable, Spot the Differences, was not included in Carroll’s analyses, but also appears to require dis-embedding. Only the sixth variable, Block Design (which was the highest loading Factor 1 variable), represents a different factor in Carroll’s analysis, namely, Visualisation. Overall, we decided that the term dis-embedding offers the best description of the demands shared by the tasks loading on Factor 1, but we understand it as broadly equivalent to the concept of Closure Flexibility (Carroll, 1993), as well as weak central coherence (Frith, 1989), and field-independence (Witkin et al., 1962). Of the seven factors identified in our analysis, the Dis-embedding factor was the only one approaching Stevens’ (1992, p. 395) criteria for a reliable factor (four or more loadings higher than .60). The remaining six factors are not considered reliable, thus their interpretation must remain tentative.

Factor 2: Global Bias. High scores on this factor represent primarily slow performance on the local level of the Navon Hierarchical Figures Test, and *accurate* performance on the global level of that test. This suggests the factor represents a general bias towards the global level of processing. This interpretation is supported by the fact that the factor is also weakly loaded with slow performance on Muller-Lyer and Good Form tasks, where slowness would indicate global bias, that is, difficulty in dis-embedding. This interpretation is inconsistent however with a weak loading the factor receives from slow performance on the Impossible-Possible Figures Test, where slowness would indicate local bias(difficulty in integration of features).

Factor 3: This factor loads substantially with accuracy of performing Muller-Lyer and Kanizsa tasks. While high accuracy on the Muller-Lyer task indicates resistance to illusion, high accuracy on the Kanizsa task indicates sensitivity to illusory contours. This factor also received a substantial loading from Muller-Lyer RT, which may indicate a strategic choice for accuracy over speed (the Muller-Lyer task produced a trade-off between accuracy and speed: High accuracy is weakly [r = .21, ns] correlated with slow performance). This factor is hard to interpret, as such we have not given it a specific label. However we tentatively suggest that it represents slow and careful task performance.

Factor 4: Kanizsa. Since high scores on this factor represent primarily low accuracy and slow speed of performing the Kanizsa task, it may be interpreted as representing task-specific competence (or, more precisely, lack of competence) on the Kanizsa task. High scores on this factor also represent low accuracy on the Spot the Differences Test and slow performance of the Good Form Task.

Factor 5: Perceptual Integration. This factor received substantial loading from Impossible Figures RT and Visual Search accuracy, and moderate loadings from the Copying and Block Design tasks. High scores on this factor represent poor, inaccurate, and slow performance on these tasks. Since all of these tasks appear to share the demand for the efficient integration of visual features, the factor may represent (poor) integration ability.

Factor 6: Cognitive Flexibility. This factor received substantial loadings from Block Design, Good Form RT, Navon local accuracy, Gestalt Completion Test, Rey figure strategy, as well as moderate loadings from the VOSP-Silhouettes and Spot the Differences Test. While these variables are heterogeneous, most seem to share the demand for dis-embedding similar to that tapped by Factor 1. Indeed, some variables load on both factors (especially Block Design), and both factors are moderately correlated (see below). Alternatively, Factor 6 could represent more general cognitive flexibility namely the ability to flexibly allocate attentional resources to optimise task performance (Booth & Happé, personal communication, February 2007).

 Factor 7: Perceptual Speed/Local Bias. This factor receives substantial loadings from Visual Search and Muller-Lyer RTs as well as the Hidden Patterns Test. Carroll (1993) identified tasks that require speed in searching for and finding or correctly comparing stimuli which can be arranged by pairs, columns, rows, or at random, as representing the factor of Perceptual Speed. This description seems to apply to our Visual Search task (where the target stimulus must be found quickly amongst the array of distractors), and Muller-Lyer task (where the rapid comparison of the length of two lines is required). Although the Hidden Patterns Test has been identified by Carroll, and in our own analysis, as representing Closure Flexibility (Dis-embedding), it also requires speeded search and comparison of stimuli, which may explain why it also loads equally strongly on Factor 7. All three variables mentioned above also appear to favour a local processing style. 

Analysis of correlations between factors (see Table 8) indicates that they are largely orthogonal. The only moderate (*r* = .41) correlation occurred between Factors 1 (Dis-embedding) and 6 (Cognitive Flexibility). Factor 1 is also weakly positively associated with Factors 3 (unnamed), 7 (Perceptual Speed / Local Bias), and 4 (Kanizsa). While the last correlation is negative it represents a positive relationship: Good ability to dis-embed (Factor 1) scores are associated with good (accurate and/or fast) performance on the variables that load onto Factor 4 (primarily Kanizsa and Spot the Differences). Additionally, Factor 7 (Perceptual Speed / Local Bias) is weakly correlated with Factors 2 (Global Bias) and 6 (Cognitive Flexibility). The first of these two correlations is negative, that is, higher perceptual speed/local bias is associated with reduced global bias.

**Table 8.** Factor Correlation Matrix of the Zero-Order Factor Analysis

Factors	1	2	3	4	5	6
1	-					
2	.04	-				
3	.27**	.16	-			
4	-.24*	-.06	-.08	-		
5	-.15	.03	-.08	-.01	-	
6	.41**	.04	.00	-.20	-.09	-
7	.23*	-.25*	-.04	-.13	-.09	.23*

*Note*. *p < .05, two-tailed. **p < .01, two-tailed.

Correlations between factor scores and the baseline/psychophysical variables (form and motion coherence thresholds, IQ, and choice RT) were generally weak (Table 9). The only moderate (.30 or above) correlations were observed between choice RT and Factors 2 (Global Bias) and 4 (Kanizsa); high scores on those factors are associated with slower choice reaction times. IQ correlated weakly with Factors 1 (Dis-embedding) and 3 (unnamed). The Motion and Form Coherence tests were threshold tests, therefore a high score represents poor performance and a low score represents good performance. The negative correlations between Factor 1 (Dis-embedding) and these tests indicate that the individuals who are good at dis-embedding tend to be good at detecting both the coherent motion and coherent form signals. The positive correlation between Factor 4 (Kanizsa) and form coherence thresholds indicates that good performance on the tasks that load onto this factor is related to good sensitivity to coherent form. The positive correlation between motion coherence thresholds and Factor 5 (Integration) indicates that good integration is related to good sensitivity to coherent motion. Finally, the negative correlation between Factor 6 (Cognitive Flexibility) and Form Coherence indicates that high cognitive flexibility is related to high sensitivity to detect coherent form. Overall, however, there is no evidence that any of the seven factors identified in our analysis reflect primarily the low level efficacy of visual perception, speed of choice reaction, or general intelligence.

**Table 9.** Factor Correlation Between Factor Scores and Background Variables

	Factor						
Variables	1	2	3	4	5	6	7
Motion Coherence	-.26*	-.12	-.11	.01	.28**	-.16	-.06
Form Coherence	-.21*	-.14	-.11	.24*	-.02	-.29**	-.17
Choice RT	-.01	.31**	-.02	.30**	.02	-.11	-.08
WASI IQ	.22*	.05	.27**	-.09	-.08	.10	.12

*Note*. Factor scores were estimated using regression method. *p < .05, two-tailed. **p < .01, two-tailed.

The preceding analyses suggested that the extracted factors represent mainly specific dimensions of visual perception, and are only weakly loaded with more general aspects of cognition (namely general intelligence or general speed of processing). However, in order to obtain the factorial structure of visual cognition that is independent from any influence of those general factors, another factor analysis was run to control for individual differences in IQ and choice RT. The second analysis was based on the matrix of standardised residuals, remaining after the scores of visual perception tests were regressed on general intelligence (WASI IQ based on two subtests) and general speed of processing (Choice RT) scores. The results were not substantially different to the factor analysis reported above and are presented in Appendix C.

Following the suggestion of one of the reviewers, we also analysed the data using the confirmatory factor analysis (CFA). Two models were tested against the data: (a) a single factor model, testing the prediction that all variables represent a single continuum of global-local perceptual bias; (b) a two factor model, testing the prediction of distinct “global” and “local” dimensions of visual perception. Individual variables were allocated to either “global” or “local” factors, depending on our analysis of the task demands. We classified the following variables as measuring global perceptual style: Gestalt Completion, VOSP silhouettes, Rey Figure strategy, Impossible Figures RT, Navon (incompatible) Global accuracy and RT, and Kanizsa accuracy and RT. The remaining variables (Block Design, Embedded Figures Task accuracy and RT, Copying, Spot the Difference, Navon [incompatible] Local accuracy and RT, Muller-Lyer accuracy and RT, Visual Search accuracy and RT, and Good Form RT) were classified as measuring local perceptual styles. The “global” and “local” factors were assumed to be correlated. The analyses were carried out using AMOS software.

For the first, single factor analysis, the CFA algorithm failed to converge at all; no solution was obtained. The second, two factor model provided a poor fit to the data (according to Blunch’s, 2008, and Byrne’s, 2001, interpretation): The parameter estimates were not statistically significant, and the fit indices were unsatisfying, χ^2^ (169) = 317.1, *p* < .001; CFI = .561; RMSEA = .099). Thus, it can be concluded that the model is considerably different from the data. While both models could be modified to improve their fit to the data (by removing certain variables from the analysis, and adding or deleting parameters), the basic fact remains: Neither a single factor nor a two factor model represent the data well – a conclusion consistent with the results of our exploratory factor analysis.

## Discussion

The aim of this study was to investigate the relationships within a set of tasks that are commonly described in the literature as measuring (weak) central coherence/field-(in)dependence (Frith, 1989, 2003; Witkin et al., 1962) as well as related, but typically poorly defined, constructs of global and local perception. Many studies have investigated these constructs, primarily in the context of autism (e.g., see Happé & Frith, 2006), but also in dyslexia (e.g., Brosnan et al., 2002; von Karolyi et al., 2003) and typical adult cognition (see Carroll, 1993). However, there have been very few attempts to clarify the relationship between these constructs, or to validate the tasks purported to operationalise them. We investigated this issue by surveying the literature on (weak) central coherence/field-(in)dependence, global and local perception; identifying a set of visual only tasks that are used to measure these constructs, and, finally, measuring the strength and direction of the relationship between them in a group of typically functioning adults.

Our search for the relevant tasks was made harder by the conceptual and terminological inconsistencies apparent in the literature. We identified the following predominant (if sometimes implicit) assumptions. The terms (weak) *central coherence* and *field-(in)dependence* are synonymous and represent the tendency to dis-embed elements from the surrounding context, and to segment local details from the global configuration. Broadly speaking, individuals who show weak central coherence/field-independence could be considered as having a locally biased perceptual style. This is in contrast to those with a more globally biased perceptual style, that is people who are strongly influenced by the surrounding context and would be described as having strong central coherence/being field-dependent.

Despite the implicit assumption within the literature that weak central coherence/field-independence is equivalent to a locally biased perceptual style and strong central coherence/field-dependence is equivalent to a globally biased perceptual style, the direct relationship between these constructs has not been examined systematically. We hypothesised that three potential outcomes were possible from our exploratory factor analyses:

1. All tasks would load on a single factor, representing a continuum of weak to strong central coherence (field-independent to field-dependent; local to global perceptual style). This factor would receive positive loading from tasks that are easier to complete for those who have a locally biased perceptual style, and negatively loaded with tasks that are easier to complete for those who have a globally biased perceptual style.

2. Local (field-independent) and global (field-dependent) tasks would load onto two separate and uncorrelated factors, indicating that local or global bias do not occur on a continuum but in fact represent independent dimensions of visual cognition.

3. The tasks would share little variance. Several different factors would emerge; they would represent some very narrow aspects of visual cognition, or be merely task-specific.

 The first hypothesis is consistent with the assumptions we identified in the literature. However the outcome of our analyses was largely consistent with the third hypothesis. It revealed that the tasks share relatively little variance – contrary to what would be expected if they measured a single construct. The factor analyses identified as many as seven factors, only one of which could be considered reliable (Stevens, 1992). This reliable first factor, which we labelled “Dis-embedding”, received substantial loading from the Block Design and Group Embedded Figures Tests. It corresponded closely to the concepts of field-independence/weak central coherence as defined by Witkin et al. (1962) and Frith (1989) which they operationalized with the Embedded Figures and Block Design tests. However, this factor captured only a relatively small proportion of overall variance, and some of the tasks that can be construed as representing weak central coherence or field-independence by virtue of a priori task analysis, and/or previous definition in the research literature (e.g., Visual Search and Muller-Lyer), did not load onto this factor. 

Factor 1 (Dis-embedding) also broadly replicated the Closure Flexibility factor identified in Carroll’s survey, defined as “speed of detecting and dis-embedding a known stimulus array from a more complex array” (Carroll, 1993, p. 341). Carroll identified Embedded Figures, Copying and Hidden Patterns among tests of closure flexibility, all of which loaded substantially on our Factor 1. Our results differed from Carroll’s in just one aspect: Whereas in our analysis the Block Design task was the highest-loading task on Factor 1, in Carroll’s analysis it belonged to a separate factor of Visualisation, defined as “the ability to comprehend imaginary movements in a 3D space, or the ability to manipulate objects in imagination” (Carroll, 1993, pp. 315-316). However, since our battery included no other tasks, apart from Block Design, that met the definition of visualisation, and since the Visualisation and Closure Flexibility factors are hard to distinguish empirically (Carroll, 1993, pp. 338-339) our outcome is not necessarily at odds with Carroll’s. Furthermore, the Block Design task, which was described very well by our seven factors (communality of over 80%), appears to have a multifactorial structure, as it loaded substantially and significantly onto two factors, and moderately on a further two.

While local perception is reasonably well represented by the tasks that load on Factor 1 (Dis-embedding) and to some degree Factor 7 (Local Bias/Perceptual Speed), the tasks that we initially identified as representing global perception do not show a clear pattern of factor loadings. Only some of these tasks were represented by our factors, and these factors (Factor 2: Global Bias and Factor 5: Integration) appear to represent different constructs. Their interpretation is far from straightforward. We interpreted Factor 2 as representing global perception, since it received loadings from variables that represented global advantage and global interference in the Navon Hierarchical Figures Test, and increased reaction time to judge line length in the Muller-Lyer illusion. In the first factor analysis, the Gestalt Completion Test also loaded weakly onto this factor, but in the second analysis this dropped out and was replaced by a globally biased Rey figure copying style. Note, however, that the positive loading of Impossible Figures RT is inconsistent with the interpretation of Factor 2 as representing global perception as high RT on this task represents reduced integrative ability. The tasks that loaded onto Factor 5 (Impossible Figures, Visual Search, Copying, Block Design) appear to require efficient integration; either of contiguous line elements (Impossible Figures), or of within-element features, for example shape and colour, as in the Visual Search Task. The interpretation of Factor 5 as representing integration, draws on previous literature (e.g., Duncan, 1995) which suggests that Visual Search requires efficient integration of features. However, this is integration in a broader sense than outlined in the introduction. Tasks that we initially identified as requiring the integration or grouping of discrete elements (e.g., Good Form Task, Gestalt Completion Test, and Kanizsa task) did not load onto Factor 5, nor onto any discrete factor which could represent global grouping or Gestalt perception.

It is important to note that the pattern of factor loadings reported here is unlikely to reflect individual differences in either IQ or general speed of making choice reactions. These two variables showed generally weak correlations with performance individual perceptual style tasks (see Table 4) and the extracted factors (see Table 9). Moreover, the second factor analysis which specifically controlled for the effects of IQ and choice RT produced results very similar to the first. Whilst we cannot be sure we eliminated Spearman’s g factor completely from our analysis, since only two tasks, Matrix Reasoning and Vocabulary, were used to measure it, we can be certain that this was not the main source of variance that was captured. What we captured was much more specific to visual perception.

The pattern of correlations and factor loadings obtained in our analyses speaks against the idea of a single continuum from global to local bias, synonymous with the continuum of central coherence or that of field dependence-independence. Our data suggest that, instead, central coherence and field dependence-independence should be understood more narrowly, as the capacity for dis-embedding only, which is not related to capacity for integration, gestalt grouping, or global perception. This outcome is consistent with some autism studies, which also demonstrate that in autism, one’s ability to dis-embed has relatively little bearing on performance on tasks that measure global perception (see Mottron, Dawson, Souliéres, Hubert, & Burack, 2006, who raise the point that enhanced local perception in autism does not necessarily imply reduced global perception in autism). While individual differences in dis-embedding ability appear to have little in common with one’s tendency towards global perception, they may be related to other factors, namely slow and careful task performance, represented on Factor 3, and cognitive flexibility, represented on Factor 6.

Given that we find support for a narrowly defined construct of weak central coherence/field-independence the question as to what underpins this construct on the psychophysiological level must be considered. In an attempt to uncover the origin of weak central coherence in autism, a range of theoretical positions have been advanced. For example, based on evidence of superior visual search for a conjunctive target in autism, it has been suggested that weak central coherence may develop from enhanced perceptual discrimination (O’Riordan & Plaisted, 2001), or enhanced perceptual functioning underpinned by over-activity in area V1 (Mottron et al., 2006). However, the implication that weak central coherence/field-independence in the typical population emerges from enhanced discrimination is not supported by the data from neuro-typical adults presented here, as performance on the Visual Search and Embedded Figures tasks were not significantly related (see Table 4; although see Jarrold et al., 2005, who have reported such a relationship in children). It has also been suggested that weak central coherence in autism emerges from reduced global grouping, specifically in the dorsal stream. This claim is based on evidence that in children with autism, performance on the Embedded Figures Test is related to ability to detect global motion (Pellicano, Gibson, et al., 2005); that is, children who are better at identifying embedded figures are less sensitive to global motion (reduced sensitivity to global motion is interpreted to reflect abnormality within the dorsal stream). However, this model is not supported by the data presented here as we found an opposite relationship: The correlation between Factor 1, representing good performance on the Group Embedded Figures Test (weak central coherence), and Motion and Form Coherence Thresholds was negative (see Table 9). The correlations between Motion or Form Coherence Thresholds and Group Embedded Figures Test accuracy were also negative (see Table 4). That is, the more field-independent the individual, the more able they were to integrate the target elements of either the motion or form signal.

In conclusion, the results of our exploratory factor analysis indicate that the 14 tasks we selected based on their use in the literature for measuring (weak) central coherence/field-(in)dependence or global/local perceptual style do not measure a unitary construct. However, we did find evidence in favour of the existence of a relatively narrow factor that represents individual differences in the ability to dis-embed relevant visual stimuli – the construct that largely corresponds to the notion of weak central coherence/field-independence, and partly also to the concept of Closure Flexibility (Carroll, 1993). In contrast, global grouping as defined by the ability to pull detached elements into a coherent whole was not represented by a single factor. Indeed, both task analysis and the outcome of the factor analysis suggests that multiple processes are involved in perceptual integration. Given that there was no significant relationship between Factor 1 and Factors 2 and 5, our results suggest that dis-embedding (or weak central coherence or field-independence) does not predict reduced global perception.

While the conclusions reached here apply directly to the adult neuro-typical population only, they may have implications for the studies of cognitive development and developmental disorders, especially autism. This is definitely so if we assume that central coherence/field dependence is a general characteristic of human cognition, and that individuals with autism represent the tail end of the normal distribution of that characteristic; that is they are different from neuro-typical individuals in degree rather than kind. If this is the case, then studies of the autism population would be expected to reveal a similar pattern of correlations to the one observed here. An alternative possibility is that all individuals with autism (or a subgroup of individuals) are qualitatively different in their cognitive skills either because of some specific deficit, or enhancement (e.g., Caron et al. 2006). If the latter is the case, then the data from our neuro-typical population may not be extrapolated easily to the autism population and much stronger associations between the tasks may, or may not, be apparent within individuals with autism. The current study cannot speak to this directly. However, it definitely makes the case for methodological caution: It is unsafe to operationalise the concepts of global and local perceptual styles purely on the basis of a priori task analysis, without empirical validation. Indeed, even defining these concepts precisely requires such validation.

## Footnotes

1 The Visual Search Task was an exception as the stimuli were either red or green. In this case the task was not completed by the colour blind participant.

## Appendix A. Descriptive statistics

**Table:**

Measure	*M*	*Mdn*	*SD*	Min-Max	*N*
Embedded Figures – accuracy	15,14	16,5	3,65	3-18	88
Embedded Figures – time (seconds)	462,17	465,5	98,83	207-600	88
Hidden Patterns – accuracy	82,25	83	18,5	43-131	89
Gestalt Completion – accuracy	14,21	15	2,67	4-19	89
Copying – accuracy	20,06	19	7,17	5-42	89
Silhouettes – accuracy	21,58	21	3,28	12-28	89
Spot the Differences – accuracy	15,51	16	4,93	2-28	88
Rey figure – copying strategy	13,69	14	4,11	5-19	75
(Navon) HFT global incompatible acc.	21,26	22	2,31	13-24	88
(Navon) HFT global incompatible RT (ms)	588,62	560	161,75	383-1683	88
(Navon) HFT local incompatible acc.	16,18	17	4,73	5-24	88
(Navon) HFT local incompatible RT (ms)	726,14	694,5	144,55	442,5-1185	88
Muller-Lyer illusory condition – accuracy	13,10	13	4,29	3-23	86
Muller-Lyer illusory condition RT (ms)	1546,63	1476,5	550,32	704-3138	86
Kanizsa illusion – accuracy	49,92	51	4,43	25-54	86
Kanizsa illusion – RT (ms)	698,33	677,3	135,57	415-1271	86
Visual Search (25 distractors) – accuracy	8,68	9	1,27	4-10	81
Visual Search (25 distractors) – RT (ms)	1056,22	1036	249,84	551-1694	81
Impossible Figures – RT	1955,40	1551	1354,48	646-7094,5	88
Good Form (orthogonal experimental – RT (ms)	591,95	561,25	134,19	424,5-1139	86
Choice RT (ms)	372,92	373	46,56	261-545,6	87
Motion Coherence (% threshold)	7,82	7,25	3,05	3,27-21,63	88
Form Coherence (% threshold)	20,74	20,72	4,11	12,87-31,5	88
Wechsler Abbreviated Scale of Intelligence					
Vocabulary – raw score	66,40	68	7,53	41-79	88
Vocabulary – T-score	63,43	66	8,50	33-76	88
Block Design – raw score	60,44	62,5	8,32	38-71	88
Block Design – T-score	61,08	62	5,70	47-69	88
Similarities – raw score	40,26	41	3,77	30-48	88
Similarities – T-score	59,05	60	6,61	41-72	88
Matrix Reasoning – raw score	29,85	30	2,82	20-35	88
Matrix Reasoning – T-score	58,18	59	5,67	40-69	88
Verbal IQ	119,18	121	11,87	86-140	88
Performance IQ	116,39	118	9,24	93-136	88
General IQ based of 4 subtests	119,84	120	9,30	100-137	88
General IQ based of 2 subtests	119,28	118	9,73	90-136	88

## Appendix B. Preliminary task analysis

**Table:**

Task and effects	Statistical analyses and their results	
**Navon Hierarchical Figures Task**	2 x 3 repeated measures ANOVA: Hierarchical Level (local or global) x Stimulus Type (compatible, neutral, or incompatible)	

Accuracy		
Main effect of hierarchical level	Higher when the target appeared at the global level	*F*(1, 87) = 89.9, *p* < .01
Main effect of stimulus type	Compatible > Neutral > Incompatible	*F*(2, 174) = 187.7, *p* < .01
Interaction	The relative disadvantage caused by incompatible stimuli was greater when the target appeared at the local level	*F*(2, 174) = 51.7, *p* < .01

Response time		
Main effect of hierarchical level	Quicker when the target appeared at the global level	*F*(1, 87) = 132.7, *p* < .01
Main effect of stimulus type	Compatible < Neutral < Incompatible	*F*(2, 174) = 153.6, *p* < .01
Interaction	The relative disadvantage caused by incompatible stimuli was greater when the target appeared at the local level	*F*(2, 174) = 14.9, *p* < .01

**Muller-Lyer Illusion Task**	Paired sample *t*-test comparing the illusory and non-illusory conditions	
Accuracy	Higher in the non-illusory condition	*t*(85) = 10.2, *p* < .05
Response time	Quicker in the non-illusory condition	*t*(85) = -20.1, *p* < .01

**Kanizsa Illusory Contour Task**	2 x 3 repeated measures ANOVA: Condition (experimental or control) x Angle of Inducer (5, 10, or 15°)	
Accuracy		
Main effect of condition	Higher in the control condition	*F*(1, 87) = 19.9, *p* < .01
Main effect of angle of inducer	15° > 10° = 5°	*F*(2, 174) = 21.1, *p* < .01
Interaction	Effect of angle of inducer seen in illusory block only	*F*(2, 174) = 7.9, *p* < .01
Response time		
Main effect of condition	Quicker in the control condition	*F*(1, 87) = 167, *p* < .01
Main effect of angle of inducer	5° < 10° = 15°	*F*(2, 174) = 42.5, *p* < .01
Interaction	Effect of angle of inducer seen in illusory block only	*F*(2,174) = 4.5, *p* < .05

**Visual Search Task**	2 x 3 repeated measures ANOVA: Target Presence (present or absent) x Set Size (5, 15, or 25)	
Accuracy		
Main effect of target presence	Higher when target was present	*F*(1, 80) = 70.8, *p* < .01
Main effect of set size	5 > 15 > 25	*F*(2, 160) = 10.1, *p* < .01
Interaction	Performance decreased as the set size increased in target present condition only	*F*(2, 160) = 12.2, *p* < .01
Response Time		
Main effect of target presence	Quicker when target was present	*F*(1, 80) = 241.5, *p* < .01
Main effect of size	5 < 15 < 25	*F*(2, 160) = 304, *p* < .01
Interaction	Response times increased as set size increased in both conditions, but the effect was larger in the target absent condition	*F*(2, 160) = 71.5, *p* < .01

**Impossible Figures Tests**^a^	Paired sample *t*-test comparing the possible and impossible trials	
Response time	Quicker when figures were possible	*t*(87) = 6.16, *p* < .01

Good form task^a^	2 x 3 repeated measures ANOVA: Condition (experimental or control) x Block Type (Simple 1, Simple 2, or Orthogonal)	
Response time		
Main effect of condition	Quicker in the control condition	*F*(1, 84) = 66.5, *p* < .01
Main effect of block type	Simple 1 = Simple 2 < Orthogonal	*F*(2, 168) = 79.5, *p* < .01
Interaction	Effect of block type significant in experimental condition only	*F*(2,168) = 38.4, *p* < .01

**Rey-sterrieth omplex Figure**	Paired samples t-tests comparing strategy score for copying and recall	*t*(88) = 16.1, *p* < .01
	Correlation between copy and recall accuracy	*r*(88) = .23, *p* < .05
	Correlation between copying strategy and recall accuracy	*r*(88) = .42, *p* < .05

*Note*. ^a^Accuracy analyses are not presented due to the majority of participants performing at ceiling in these tasks.

## Appendix C. Factor analysis of the standardized residuals mamatrix

Preliminary analyses established that the data were suitable for the factor analysis (Bartlett’s Sphericity test, χ^2^ = 498.247, *df* = 210, *p* < .001; determinant of correlation matrix = .002; Kaiser-Meyer-Olkin measure of sampling adequacy = .654). These values are very similar to those obtained in the previous analysis, indicating once more a relatively diffuse pattern of correlations. The analysis of anti-image correlation matrix diagonals brought unsatisfactory results (< .50) for Navon global RT. This variable was excluded from the analysis, which resulted in the improvement of sampling adequacy (Kaiser-Meyer-Olkin = .701).

As in the previous analysis, Kaiser’s criterion of eigenvalues greater than 1 resulted in extraction of 7 factors. Together they explained 44.6% of total variance. Most communality values were again lower than .50. Only Block Design showed a high communality value (above .80; see Table C1). Coefficients of regression of variables on factors (pattern matrix) and factor loadings, that is coefficients of correlations between variables and factors (structure matrix) are presented in Tables C2 and C3, respectively.

The factorial solution obtained for the correlation matrix of standardised residuals was very similar to that obtained previously for the correlation matrix of raw scores. Correlations between corresponding factors scores from both analyses (estimated using regression methods) were very high (*r* = .90 - .99). Note, however, that Factor 6 (Cognitive Flexibility) obtained in the previous analysis now appears as Factor 7 (and vice versa). Also the vector of Factor 7 (previously 6) is reversed, that is, high scores on this factor now represent low cognitive flexibility.

Table C4 shows that the matrix of correlations between factors is similar to that obtained in the previous analysis. Good ability to dis-embed (Factor 1) is moderately related to high cognitive flexibility (Factor 7, previously 6), and weakly related to reduced global bias (Factor 4) and high perceptual speed/local bias (Factor 6, previously 7). Moreover, high perceptual speed/local bias is weakly related to high cognitive flexibility.

**Table C1.** Communalities in the Second Factor Analysis

Variables	Initial	Extraction
Block Design	.698	.822
Group Embedded Figures Test accuracy	.522	.605
Group Embedded Figures Test RT	.401	.426
VOSP-Silhouettes	.201	.201
Gestalt Completion Test	.329	.330
Hidden Patterns Test	.315	.394
Copying Test	.366	.379
Spot the Differences Test	.408	.461
Rey figure: Copying strategy	.300	.400
Impossible Figures RT	.286	.488
Muller-Lyer illusory condition accuracy	.266	.431
Muller-Lyer illusory condition RT	.464	.654
Visual Search, target present within 25 distractors accuracy	.222	.290
Visual Search, target present within 25 distractors RT	.279	.374
Kanizsa illusory condition accuracy	.409	.682
Kanizsa illusory condition RT	.224	.281
Good Form experimental orthogonal RT	.430	.469
Navon Hierarchical Figures Test, incompatible, global acc	.333	.360
Navon Hierarchical Figures Test, incompatible, local acc	.305	.314
Navon Hierarchical Figures Test, incompatible, local RT	.370	.562

**Table C2.** Pattern Matrix of the Second Factor Analysis

Variables	Factors						
	1	2	3	4	5	6	7
Embedded Figures acc	.783						
Embedded Figures RT	-.588						
Block Design	.553						-.384
Copying	.439				-.312		
Navon local RT		.742					
Navon global acc		.452					
Muller-Lyer acc			.611				
Kanizsa acc				-.729			
Kanizsa RT				.388			
Spot the Differences	.375			-.380			
Impossible Figures RT					.581		
Visual Search acc					-.390		
Visual Search RT						-.610	
Muller-Lyer RT			.431			-.521	
Hidden Patterns						.449	
Gestalt Completion							-.548
Navon local acc							-.447
Good Form RT			.387				.423
Rey figure strategy		.398					-.420
VOSP Silhouettes							

*Note*. Coefficients are sorted by size. Those lower than .30 are not displayed.

**Table C3.** Structure Matrix of the Second Factor Analysis

Variables	Factors						
	1	2	3	4	5	6	7
Block Design	.768				-.370	.357	-.685
Embedded Figures acc	.752						
Embedded Figures RT	-.620						.318
Copying	.519				-.388		-.323
Spot the Differences	.516			-.509			-.328
Navon local RT		.732					
Navon global acc		.509		-.331			
Muller-Lyer acc			.575				
Kanizsa acc			.358	-.761			
Kanizsa RT				.369			
Impossible Figures RT		.335			.570		
Visual Search acc					-.386		
Muller-Lyer RT		.375	.546			-.624	
Visual Search RT						-.588	
Hidden Patterns	.401					.512	
Gestalt Completion							-.527
Good Form RT	-.316		.391				.525
Navon local acc	.356						-.523
Rey figure strategy		.360					-.476
VOSP-Silhouettes							-.353

*Note*. Factor loadings are sorted by size. Those lower than .30 are not displayed. Loadings that are statistically significant according to Stevens' (1992) interpretation are highlighted.

**Table C4.** Factor Correlation Matrix of the Second Factor Analysis

Factors	1	2	3	4	5	6
1						
2	.06					
3	.13	.11				
4	-.25*	-.14	-.08			
5	-.15	.04	-.06	-.01		
6	.24*	-.20	-.15	-.07	-.12	
7	-.42**	-.07	.05	.14	.13	-.28**

*Note*. **p* < .05, two-tailed. ***p* < .01, two-tailed.
